# Association of triglyceride-glucose index with atherosclerotic cardiovascular disease and mortality among familial hypercholesterolemia patients

**DOI:** 10.1186/s13098-023-01009-w

**Published:** 2023-03-09

**Authors:** Jun Wen, Qi Pan, Lei-Lei Du, Jing-Jing Song, Yu-Peng Liu, Xiang-Bin Meng, Kuo Zhang, Jun Gao, Chun-Li Shao, Wen-Yao Wang, Hao Zhou, Yi-Da Tang

**Affiliations:** 1 Department of Cardiology, State Key Laboratory of Cardiovascular Disease, National Center for Cardiovascular Diseases, Fuwai Hospital, Chinese Academy of Medical Sciences and Peking Union Medical College, Beijing, China; 2 Department of Cardiology and Institute of Vascular Medicine, Key Laboratory of Molecular Cardiovascular Science, Ministry of Education, Peking University Third Hospital, No.49 Huayuanbei Road, Beijing, 100191 China; 3 Department of Cardiology, The First Affiliated Hospital of Wenzhou Medical University, NanBai Xiang Avenue, Ouhai District, Wenzhou, 325000 China; 4 Department of Cardiology, Guangdong Cardiovascular Institute, Guangdong Provincial People’s Hospital, Guangdong Academy of Medical Sciences, Guangzhou, China

**Keywords:** Triglyceride glucose index, Familial hypercholesterolemia, Insulin resistance, Atherosclerotic cardiovascular disease, All-cause mortality

## Abstract

**Background:**

Familial hypercholesterolemia (FH) is an inherited metabolic disorder with a high level of low-density lipoprotein cholesterol and the worse prognosis. The triglyceride-glucose (TyG) index, an emerging tool to reflect insulin resistance (IR), is positively associated with a higher risk of atherosclerotic cardiovascular disease (ASCVD) in healthy individuals, but the value of TyG index has never been evaluated in FH patients. This study aimed to determine the association between the TyG index and glucose metabolic indicators, insulin resistance (IR) status, the risk of ASCVD and mortality among FH patients.

**Methods:**

Data from National Health and Nutrition Examination Survey (NHANES) 1999–2018 were utilized. 941 FH individuals with TyG index information were included and categorized into three groups: < 8.5, 8.5–9.0, and > 9.0. Spearman correlation analysis was used to test the association of TyG index and various established glucose metabolism-related indicators. Logistic and Cox regression analysis were used to assess the association of TyG index with ASCVD and mortality. The possible nonlinear relationships between TyG index and the all-cause or cardiovascular death were further evaluated on a continuous scale with restricted cubic spline (RCS) curves.

**Results:**

TyG index was positively associated with fasting glucose, HbA1c, fasting insulin and the homeostatic model assessment of insulin resistance (HOMA-IR) index (all *p* < 0.001). The risk of ASCVD increased by 74% with every 1 unit increase of TyG index (95%CI: 1.15–2.63, *p* = 0.01). During the median 114-month follow-up, 151 all-cause death and 57 cardiovascular death were recorded. Strong U/J-shaped relations were observed according to the RCS results (*p* = 0.0083 and 0.0046 for all-cause and cardiovascular death). A higher TyG index was independently associated with both all-cause death and cardiovascular death. Results remained similar among FH patients with IR (HOMA-IR ≥ 2.69). Moreover, addition of TyG index showed helpful discrimination of both survival from all-cause death and cardiovascular death (*p* < 0.05).

**Conclusion:**

TyG index was applicable to reflect glucose metabolism status in FH adults, and a high TyG index was an independent risk factor of both ASCVD and mortality.

## Introduction

Atherosclerosis cardiovascular disease (ASCVD) is the leading cause of death in both developed and developing countries [1]. Atherosclerotic plaque formation and development is the most important pathophysiological process in ASCVD, which is associated with endothelial cell injury, inflammation, oxidative stress, lipid and other metabolic alterations, and thrombosis [2, 3]. Familial hypercholesterolemia (FH) is an inherited metabolic disorder resulting in lifetime exposure to high levels of low-density lipoprotein cholesterol (LDL-C) and consequently, an elevated risk of ASCVD [4, 5]. However, despite the heavy cardiovascular metabolic burden, the prevalence of type 2 diabetes (T2D), another common risk factor of ASCVD, is lower in FH patients compared with unaffected relatives [6] or the normal population [7, 8]. Given the idea, the glucolipid metabolism of FH patients seems to be different from normal persons, although there has not been convincing explanation. Besides, the combination of FH and T2D doubles the risk of cardiovascular disease in persons with FH [7, 8]. Given the glucolipid discord and the detrimental synergistic effects, it’s essential to verify the efficacy of established glucolipid metabolism-related biomarkers and develop novel biomarkers among FH patients [9].

The triglyceride-glucose (TyG) index, calculated as Ln (fasting triglycerides [TG, mg/dl] × fasting blood glucose [mg/dl]/2), is an emerging tool to reflect insulin resistance (IR) [10]. IR is the earlier stage and principal characteristic of T2D and also leads to a cluster of abnormalities including accelerated atherosclerosis, hypertension or polycystic ovarian syndrome [11, 12]. Recent studies revealed the relationship between TyG index and pro-atherosclerotic factors such as inflammation, endothelial dysfunction, glucolipid metabolism disorders and thrombosis [13–15]. Therefore, it’s not surprising that the TyG index was positively associated with a higher prevalence of a series of diseases such as symptomatic coronary artery disease and all-cause mortality [10, 16]. However, the effects of IR (refer to higher TyG index) on cardiovascular health, and the value of TyG index to reflect IR and glucose metabolic status as well as predict ASCVD and mortality risks in FH patients has not been evaluated. In view of the above, data from a nationally representative sample of FH individuals from National Health and Nutrition Examination Surveys (NHANES) were utilized to determine the association between the TyG index and glucose metabolic indicators, IR status, the risk of ASCVD and mortality among FH adults.

## Methods

### Study population

NHANES is a two-year-cycle cross-sectional survey conducted by the Centers for Disease Control and Prevention (CDC) of America, involving a home interview and a medical examination, offering demographics, socioeconomic status, dietary and health information as well as physical and physiological measurements of U.S. population [17]. NHANES study protocol was approved by The National Center for Health Statistics (NCHS) ethics committee (Protocol #2011–17, https://www.cdc.gov/nchs/nhanes/irba98.htm). Informed consent from all the participants was obtained before participating.

**Fig. 1:**
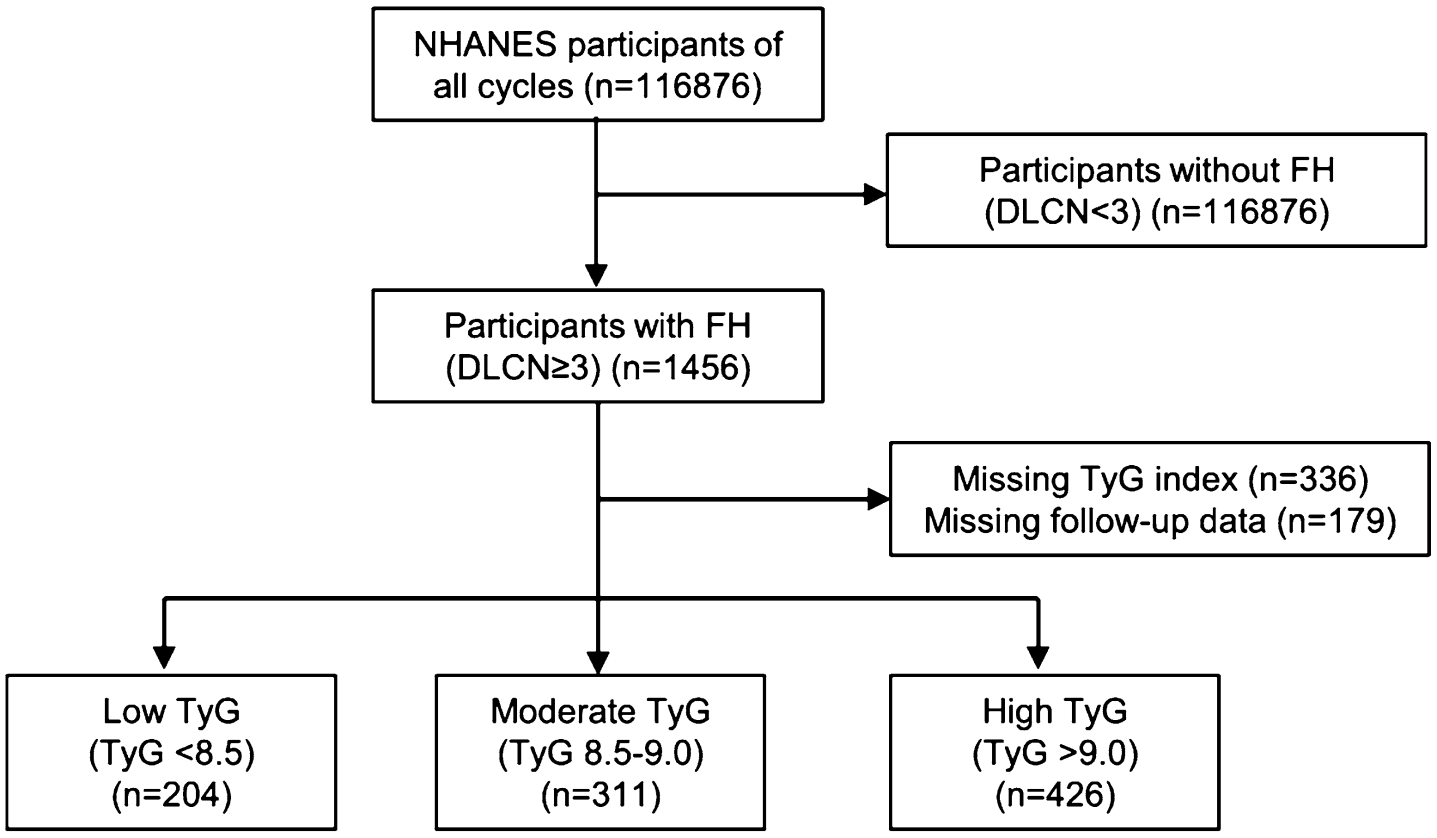
Flowchart of the study population

There were 1456 out of 116876 participants in the survey diagnosed as FH in accordance with a Dutch Lipid Clinical Network (DLCN) index that was higher than or equal to 3 points, as described previously [18]. Among them, TyG index was available in 1120 individuals (n = 336 excluded). Participants (n = 179) were excluded due to loss of follow-up. Therefore, a total of 941 individuals (530 females and 411 males), is the final study population (Fig. 1). The study population was divided into 3 groups: < 8.5, 8.5–9.0, > 9.0 to make the participants number in the groups comparable, as reported previously [19].

### Data collection

Information on socioeconomic conditions, behavior and history of diseases was obtained through questionnaires by experienced interviewers. Drinking was defined as having at least 12 alcohol drinks per year; smoking was defined as having smoked ≥ 100 cigarettes in life [20]. History of ASCVD, hypertension or diabetes was defined as self-reported physician diagnosis [21]. History of ASCVD was defined as self-reported physician diagnosis. “Has a doctor or other health professional ever told {you/SP} that {you/s/he} had a coronary heart disease/angina, also called angina pectoris/heart attack (also called myocardial infarction)/stroke?” was a question on the medical conditions section of the household questionnaires via home interview, and those who answered “yes” were deemed to have a history of ASCVD. Body mass index (BMI) was calculated using weight (kg)/height (m2). Income status of the family was described with poverty-income ratio, which was the ratio of family income to the poverty threshold. TyG index was calculated as Ln (fasting triglycerides [TG, mg/dl] × fasting blood glucose [mg/dl]/2) [10]. Laboratory results were obtained from serum specimens when they visited the mobile examination center and vials were stored under appropriate frozen (− 30 °C) conditions until they were shipped to National Center for Environmental Health for testing [22]. The original homeostatic model assessment insulin resistance index (HOMA-IR) was calculated as fasting insulin × fasting blood glucose/22.5 [23]. Homeostatic model assessment insulin sensitivity index (HOMA-IS) was calculated as 1/HOMA-IR. Details for each variable measurement is published on the NHANES website [24].

### Follow-up and endpoints

The period of follow-up lasted from the date of the interview through the last follow-up time, Dec 31 2019, or the date of death, whichever came first. Records from the NDI provided information on these including participants' causes of death. The endpoints for this study included all-cause mortality and cardiovascular death, which encompassed cardiac death (e.g., sudden cardiac death and myocardial infarction) and vascular death (e.g., stroke) [1]. The median follow-up duration is 114 months (interquartile range, 57–159 months). The maximum follow-up duration is 248 months.

### Statistical analysis

Data were analyzed using SPSS complex sample module version 22.0 (IBM Corp, Armonk, NY) and R (version 4.2.2). The Kolmogorov–Smirnov normality test was adopted to test the normality of continuous variables. Normally distributed variables were described with mean ± standard deviation (SD). Variance analysis was adopted to compare the mean levels while Chi-square tests were chosen to compare the percentages of categorical variables across the different groups.

Spearman correlation analysis was used to test the association of TyG index and various established glucose metabolism-related indicators (fasting blood glucose, fasting insulin, HbA1c, HOMA-IR and HOMA-IS index. Both univariable and multivariable-adjusted logistic regression were used to calculate the odds ratio (OR) with 95% confidence interval (CI) for the relationship between TyG index and ASCVD. The possible nonlinear relationships between TyG index and the all-cause or cardiovascular death were further evaluated on a continuous scale with restricted cubic spline (RCS) curves based on the multivariable Cox proportional hazards models, with four nodes at the fixed percentiles of 5%, 35%, 65% and 95% of the distribution of TyG index. The event-free survival rates among the groups were estimated by the Kaplan–Meier method and compared by the log-rank test. Cox regression analysis was used to assess the association between TyG index and mortality. The following variables were utilized as covariates in the study population: age, gender, BMI, smoke, drink, HOMA-IR, low-density lipoprotein cholesterol (LDL-C), creatinine and hypertension. To assess the added prognostic value of TyG index beyond the original model, C-index was calculated, using predict.coxph function to predict Cox model with predict value type = "survival". Furthermore, a sensitivity analysis was applied to further investigate the association of TyG index with ASCVD and mortality in FH patients with IR (HOMA-IR ≥ 2.69) [25]. A two-sided p < 0.05 was considered statistically significant.

## Results

### Demographic characteristics of the study population

Among the 941 FH participants in this study, 204 (21.68%) had TyG index < 8.5 (low TyG index), 311 (33.05%) had TyG index ≥ 8.5 and ≤ 9.0 (moderate TyG index), and 426 (45.27%) had TyG index > 9.0 (high TyG index; Table 1). As expected, those with higher TyG index had higher levels of fasting blood glucose, fasting total glyceride (TG), fasting insulin, HbA1c and HOMA-IR index (all *p* < 0.001), in addition to higher occurrence rate of diabetes and impaired fasting blood glucose (IFG; *p* < 0.001) than those with lower TyG index. Besides, individuals with higher TyG index were also more likely to be older, white and had higher BMI, systolic blood pressure (SBP), fasting total cholesterol (TC) and LDL-C as well as lower high-density lipoprotein cholesterol (HDL-C; all *p* < 0.05) compared with those with relatively lower TyG index. There was no significant difference among the four groups in DLCN score, poverty-income ratio, drink, smoke, hypertension history, diastolic blood pressure (DBP), alanine aminotransferase (ALT), aspartate aminotransferase (AST) as well as serum creatinine (p > 0.05).

**Table 1:** Demographic characteristics of the study population

	TyG < 8.5 (N = 204)	TyG 8.5–9 (N = 311)	TyG > 9 (N = 426)	*p* value
Age (SD) (y.o.)	50.0 (15.5)	50.6 (16.0)	53.6 (14.4)	0.004
Female (%)	125 (61.3%)	164 (52.7%)	241 (56.6%)	0.159
Race (%)				< 0.001
White	89 (43.6%)	163 (52.4%)	228 (53.5%)	
Black	64 (31.4%)	62 (19.9%)	57 (13.4%)	
Mexican American	13 (6.37%)	40 (12.9%)	70 (16.4%)	
Other	38 (18.6%)	46 (14.8%)	71 (16.7%)	
BMI (SD) (kg/m2)	28.6 (7.37)	29.3 (6.31)	30.4 (6.60)	0.004
Poverty ratio (SD)	2.46 (1.65)	2.29 (1.56)	2.34 (1.55)	0.504
DLCN score (SD)	3.51 (0.79)	3.57 (0.84)	3.61 (0.99)	0.397
DM status (%)				< 0.001
DM	16 (7.92%)	38 (12.8%)	159 (39.2%)	
IFG	12 (5.94%)	32 (10.7%)	55 (13.5%)	
IGT	11 (5.45%)	26 (8.72%)	27 (6.65%)	
No	163 (80.7%)	202 (67.8%)	165 (40.6%)	
Education status (%)				0.005
College or above	108 (53.2%)	147 (47.4%)	165 (38.9%)	
High school or equivalent	50 (24.6%)	72 (23.2%)	111 (26.2%)	
Less than high school	45 (22.2%)	91 (29.4%)	148 (34.9%)	
Drink (%)	132 (64.7%)	187 (60.1%)	235 (55.2%)	0.064
Smoke (%)	107 (52.7%)	176 (57.5%)	246 (57.7%)	0.748
ASCVD (%)	59 (29.1%)	89 (29.1%)	146 (34.4%)	0.126
Hypertension (%)	98 (48.0%)	145 (46.6%)	223 (52.3%)	0.275
SBP (SD) (mmHg)	126 (23.0)	125 (19.3)	129 (20.7)	0.025
DBP (SD) (mmHg)	71.8 (13.0)	70.7 (12.6)	72.1 (13.6)	0.388
HbA1c (SD) (%)	5.5 (0.5)	5.7 (0.7)	6.5 (2.0)	< 0.001
Fasting insulin (μU/mL)	9.15 (6.45)	13.9 (28.7)	16.6 (17.3)	< 0.001
Fasting glucose (SD) (mmol/L)	5.35 (0.66)	5.59 (0.93)	7.32 (3.56)	< 0.001
HOMA-IR index (SD)	2.22 (1.70)	3.52 (7.29)	5.61 (8.81)	< 0.001
ALT (SD) (U/L)	23.9 (15.7)	26.8 (27.0)	34.2 (96.5)	0.139
AST (SD) (U/L)	26.4 (21.6)	26.8 (31.2)	27.0 (18.7)	0.952
Creatinine (SD) (μmol/L)	76.1 (22.3)	75.8 (26.3)	77.8 (32.4)	0.593
Fasting TG (SD) (mg/dL)	79.3 (18.6)	128 (24.9)	216 (72.1)	< 0.001
Fasting TC (SD) (mg/dL)	255 (56.5)	262 (53.0)	280 (60.6)	< 0.001
HDL-C (SD) (mg/dL)	61.2 (17.9)	52.3 (13.6)	47.9 (13.7)	< 0.001
LDL-C (SD) (mg/dL)	178 (53.3)	184 (49.0)	190 (55.7)	0.038

TyG, triglyceride-glucose index; SD, standard deviation; BMI, body mass index; DLCN, Dutch Lipid Clinical Network; DM, diabetes mellitus; IFG, impaired fasting blood glucose; IGT, impaired glucose tolerance; ASCVD, atherosclerotic cardiovascular diseases; DBP, diastolic blood pressure; SBP, systolic blood pressure; ALT, alanine aminotransferase; AST, aspartate aminotransferase; TG, total glyceride; TC, total cholesterol; HDL-C, high density lipoprotein cholesterol; LDL-C, low density lipoprotein cholesterol

### Correlation between TyG index and established glucose metabolism indicators among FH patients

As a novel indicator of IR, the diagnostic value of TyG index in FH population was examined by assessing the association with those well-recognized indicators reflecting glucose metabolism status including fasting blood glucose, HbA1c, fasting insulin, HOMA-IR and HOMA-IS. As shown in Table 2, TyG index was positively associated with fasting glucose, HbA1c, fasting insulin and HOMA-IR index, and negatively associated with HOMA-IS (all *p* < 0.001) among FH patients, indicating that TyG index was applicable to FH individuals to reflect the glucose metabolism status.

**Table 2:** Correlation between TyG index and established glucose metabolism-related indicators

Variables	Correlation coefficient (*r*)	*p* value
Fasting glucose (mmol/L)	0.462	< 0.001
HbA1c (%)	0.333	< 0.001
Fasting insulin (μU/mL)	0.272	< 0.001
HOMA-IR	0.407	< 0.001
HOMA-IS	-0.407	< 0.001

HOMA-IR, homeostatic model assessment insulin resistance index; HOMA-IS, homeostatic model assessment insulin sensitivity index

### Association of TyG index with ASCVD among FH patients

Given the idea that TyG index is associated with the development and prognosis of cardio-cerebrovascular diseases [26, 27], we evaluated the relationship in FH population by conducting a multivariable-adjusted logistic analysis (Table 3). After adjustment with age, gender, BMI, smoke, drink, HOMA-IR, LDL-C, creatinine and hypertension, the OR was 2.38 (95% CI 1.25–4.53, *p* = 0.01) in the group with high TyG index compared with the group with low TyG index; while no significant difference was observed between the group with moderate and low TyG index (OR, 1.45; 95%CI 0.73–2.89, *p* = 0.29). For every 1 unit increase of TyG index, the risk of ASCVD increased by 74% after adjustment (95%CI 1.15–2.63, p = 0.01). In the sensitivity analysis, TyG index remained significantly associated with ASCVD in FH patients with IR (Crude OR, 1.52, 95%CI 1.09–2.13, *p* = 0.02; Adjusted OR, 2.14; 95%CI 1.19–3.86, *p* = 0.01). These results indicated that increase of TyG index was independently associated with the elevation of ASCVD risk among FH adults.

**Table 3:** Odd ratios of TyG index with ASCVD among FH participants

	NO. Events/subjects	Crude OR	*p* value	Adjusted OR	*p* value
Low TyG (< 8.5)	59/204	Ref		Ref	
Moderate TyG (8.5–9.0)	89/311	0.94 (0.67–1.46)	0.94	1.45 (0.73–2.89)	0.29
High TyG (> 9.0)	146/426	1.28 (0.89–1.84)	0.18	**2.38 (1.25–4.53)**	**0.01**
All FH participants					
Every 1 unit increase of TyG	294/941	**1.26 (1.02–1.57)**	**0.03**	**1.74 (1.15–2.63)**	**0.01**
FH participants with IR					
Every 1 unit increase of TyG	166/458	**1.52 (1.09–2.13)**	**0.02**	**2.14 (1.19–3.86)**	**0.01**

OR, odd ratios; Ref. reference, IR, insulin resistance

Adjusted model: age, gender, BMI, smoke, drink, HOMA-IR, LDL-C, creatinine and hypertension

### Association of TyG index with mortality among FH patients

**Fig. 2:**
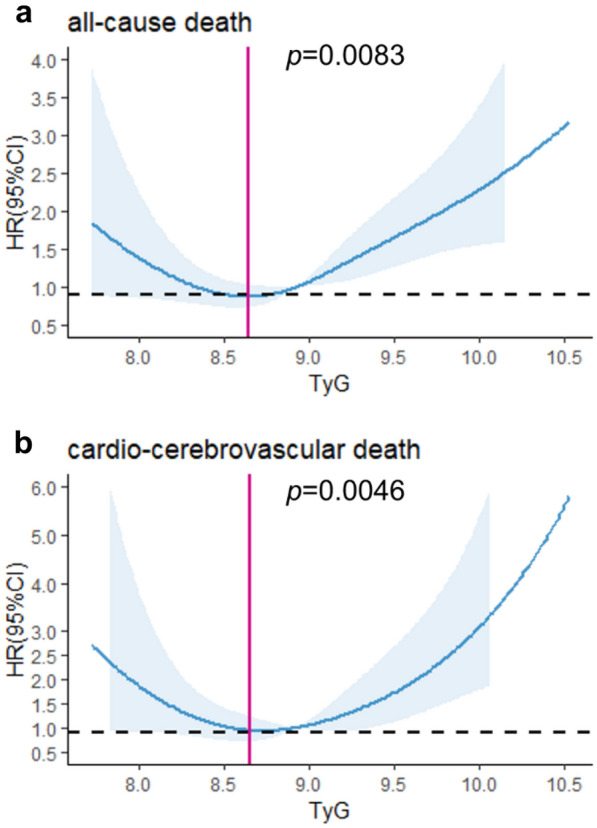
Restricted cubic spline curves (RCS) for the relationship between TyG index and the all-cause death **A** and cardiovascular death **B**

**Fig. 3:**
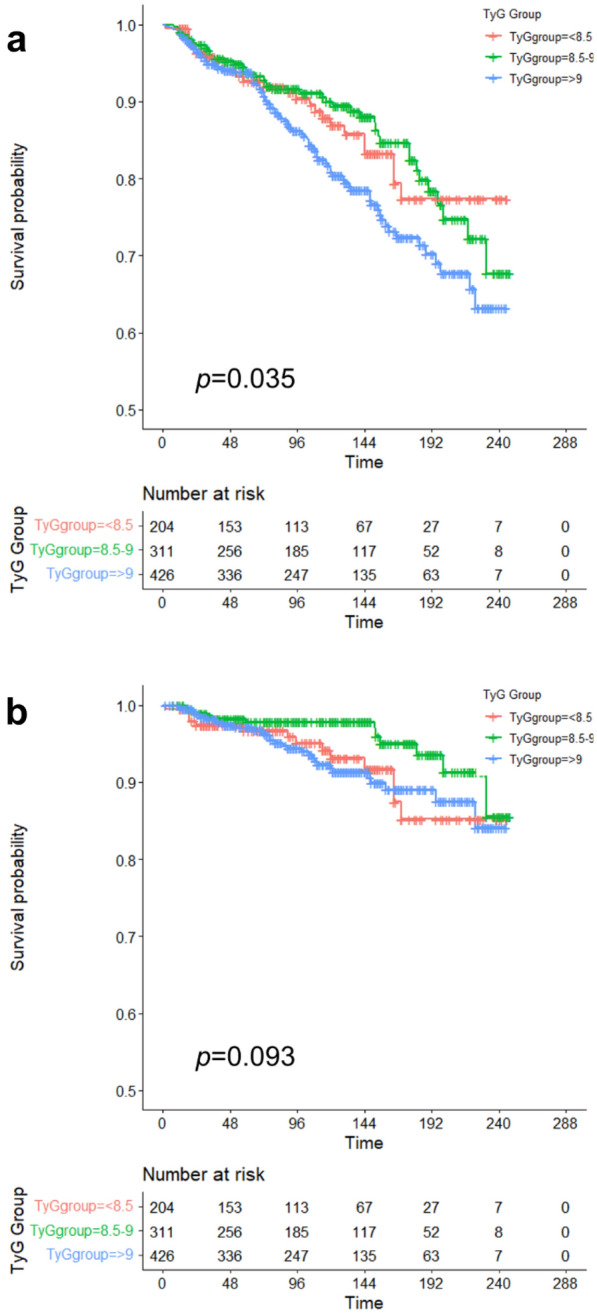
Cumulative hazard of all-cause death **A** and cardiovascular death **B** across the groups with different TyG index

In this study, the median follow-up duration is 114 months (interquartile range, 57–159 months). In Fig. 2, we used RCS to flexibly model and visualize the relationship with all-cause and cardiovascular mortality in FH adults. The risk of both all-cause and cardiovascular mortality was relatively flat until around 8.6 of TyG index, and then started to increase rapidly afterwards (*p* for non-linearity = 0.0083 and 0.0046, respectively). The study population was divided into three groups (< 8.5, 8.5–9.0 and > 9.0) due to the strong U/J-shaped relation between TyG index and mortality. Figure 3 depicts the cumulative hazard of all-cause death and cardiovascular death in the groups with different TyG index. FH patients reported > 9.0 had lower survival probability compared with those reported low or moderate TyG index (log-rank *p* = 0.035). No significant difference was observed in the incidence of cardiovascular mortality (log-rank *p* = 0.093). Cox analysis was utilized to further assess the association of TyG index with all-cause death and cardiovascular death (Table 4). After multivariable adjustment, TyG index was independently associated with both all-cause death (HR, 1.55; 95%CI 1.10–2.18, *p* = 0.01 for every 1 unit increase of TyG index) and cardiovascular death (HR, 1.79; 95%CI 1.04–3.09, *p* = 0.04 for every 1 unit increase of TyG index). In sensitivity analysis (Table 5), TyG index remained significantly associated with all-cause and cardiovascular death in FH patients with IR (HR, 1.91; 95%CI 1.12–3.24, *p* = 0.02 and HR, 2.73; 95%CI 1.11–6.74, *p* = 0.03, respectively). The adjusted HR was 1.61 (95%CI 1.07–2.42, *p* = 0.02) for all-cause death and 2.09 (95%CI 1.02–4.30, *p* = 0.045) for cardiovascular death in the group with high TyG index comparing with the group with moderate TyG index. However, no significant difference was observed when comparing the two groups reported low and moderate TyG index (HR, 1.19; 95%CI 0.71–2.00, *p* = 0.51 for all-cause death; HR, 2.19; 95%CI 0.96–4.97, *p* = 0.06 for cardiovascular death). In Cox prediction models, C-statistic values were 0.489 (95%CI 0.437–0.542) and 0.408 (95%CI 0.324–0.491) for survival from all-cause death and cardiovascular death with traditional risk factors, respectively (Table 6). Addition of TyG index to the original model resulted in significant improvements in discrimination of both survival from all-cause death (C-statistic 0.483; 95%CI 0.432–0.535, *p* = 0.042) and cardiovascular death (C-statistic 0.397; 95%CI 0.315–0.479, *p* = 0.042). These results demonstrated that TyG index was an independent risk factor for all-cause death and cardiovascular death among FH adults.

**Table 4:** Hazard ratios of TyG index with all-cause and cardiovascular mortality among FH participants

	NO. Events/subjects	Crude HR	*p* value	Adjusted HR	*p* value
**All-cause death**	**151/941**				
Low TyG (< 8.5)	26/204	1.05 (0.64–1.71)	0.85	1.19 (0.71–2.00)	0.51
Moderate TyG (8.5–9.0)	42/311	Ref		Ref	
High TyG (> 9.0)	83/426	**1.55 (1.07–2.24)**	**0.02**	**1.61 (1.07–2.42)**	**0.02**
Every 1 unit increase of TyG		**1.54 (1.16–2.06)**	**0.003**	**1.55 (1.10–2.18)**	**0.01**
**Cardiovascular death**	**57/941**				
Low TyG (< 8.5)	14/204	1.96 (0.91–4.23)	0.09	2.19 (0.96–4.97)	0.06
Moderate TyG (8.5–9.0)	12/311	Ref		Ref	
High TyG (> 9.0)	31/426	**2.02 (1.04–3.94)**	**0.04**	**2.09 (1.02–4.30)**	**0.045**
Every 1 unit increase of TyG		**1.68 (1.06–2.67)**	**0.03**	**1.79 (1.04–3.09)**	**0.04**

HR, hazard ratios

Adjusted model: age, gender, BMI, smoke, drink, HOMA-IR, LDL-C, creatinine and hypertension

**Table 5:** Hazard ratios of TyG index with all-cause and cardiovascular mortality among FH participants with IR

	NO. Events/subjects	Crude HR	*p* value	Adjusted HR	*p* value
**All-cause death**	**66/458**				
Every 1 unit increase of TyG		2.10 (1.37–3.23)	0.001	1.91 (1.12–3.24)	0.02
**Cardiovascular death**	**28/458**				
Every 1 unit increase of TyG		2.54 (1.32–4.88)	0.01	2.73 (1.11–6.74)	0.03

Adjusted model: age, gender, BMI, smoke, drink, HOMA-IR, LDL-C, creatinine and hypertension

**Table 6:** C-index of TyG for predicting all-cause and cardiovascular death in FH patients

Models	C-statistic (95% CI) *	*p* value
All-cause death		
Original model **	0.489 (0.437–0.542)	Ref
Original model + TyG	**0.483 (0.432–0.535)**	**0.042**
Cardiovascular death		
Original model	0.408 (0.324–0.491)	Ref
Original model + TyG	**0.397 (0.315–0.479)**	**0.042**

^*^The C-index was calculated by using the predict.coxph function to predict the Cox model with predicted value type = "survival"

^**^Original model included age, gender, BMI, smoke, drink, HOMA-IR, LDL-C, creatinine and hypertension

## Discussion

Herein, we combined NHANES data from 1999 to 2018, and a total of 941 FH participants with TyG index and follow-up data accessible were finally included. The capabilities of TyG index to reflect glucose metabolism status (hyperglycemia and IR) and predict the risks of ASCVD and mortality were preliminarily verified. As a cost-effective tool, TyG index integrates fasting glucose and triglycerides levels and could provide an early relevant clinical evaluation of glucolipid metabolic disorder such as IR, and potential prediction value of ASCVD and mortality risks. To the best of our knowledge, this is the first study to examine the value of TyG index among adults with FH.

As a hallmark of T2D, IR is a state of decreased sensitivity and responsiveness to the action of insulin [28]. Arguably, the gold standards of IR diagnosis are euglycemic insulin clamp and intravenous glucose tolerance testing; however, they have not been applied in clinical practice due to invasiveness and high cost [29]. TyG index is used as a novel marker of IR in healthy individuals, according to a considerable number of studies since 2014. In a Korean study with 5354 middle-aged nondiabetic individuals enrolled, the risk of diabetes onset was fourfold higher in the highest quartile compared with the lowest quartile (relative risk, 4.10; 95%CI 2.70–6.21) [30]. In a White European cohort with 4820 participants, the HR was 5.59 (95% CI 3.51–8.91) in the fourth quartile *vs*. the bottom quartile [31]. However, it’s important to note that the applicability of TyG index to detect IR among specific populations with metabolism characteristics should be further evaluated in theory because TyG index largely depends on the glucolipid metabolic status. For instance, the risk of T2D in lean Koreans increased along with the increase of TyG index with HRs of in each quartile were 1.00, 1.63 (95%CI 1.18–2.24), 2.30 (95%CI 1.68–3.14) and 3.67 (95%CI 2.71–4.98), respectively [32]. Whereas, TyG index had lower sensitivity and specificity compared with HOMA-IR, as reported in a study based on healthy Argentinean children aged 9.3 ± 2.2 years old [33]. FH is a common inherited condition leading to significant metabolism disorders, characterized by high LDL-C level. Interestingly, in spite of the heavy cardiovascular metabolic burden, the prevalence of T2D is lower in FH patients [6–8]. Furthermore, Mendelian randomization analysis suggested a significant association between gene variants determining higher LDL-C levels and a lower risk of T2D [34]. Given the glucolipid metabolism features and the high prevalence of FH (estimated at 1 in 200), efforts to investigate the capability of TyG index to evaluate glucose metabolism disorders among FH patients are required [9]. Results in this pilot study demonstrated that TyG index was positively associated with well-recognized indicators such as fasting blood glucose (*r*, 0.462; *p* < 0.001), HbA1c (*r*, 0.333; *p* < 0.001), fasting insulin (*r*, 0.272; *p* < 0.001) and HOMA-IR (*r*, 0.407; *p* < 0.001) in FH population. Therefore, TyG index seems to be applicable to FH patients.

Despite major advances in understanding of the disease and effective therapies such as lipid-lowering drugs and dietary interventions, FH is still underdiagnosed and undertreated [35]. As a result, FH is an important risk factor of ASCVD and premature deaths [36]. IR and T2D have also been reported to increase the risk of ASCVD by exerting harmful effects on the vascular smooth muscle cells, macrophages and endothelium [37]. The effects of T2D on the risk of cardiovascular disease in FH patients were evaluated by Climent et al. where the OR was 2.01 (95%CI 1.18–3.43, *p* = 0.01), suggesting T2D and IR led to additional ASCVD risk [7]. As a result, it’s essential to develop reliable and convenient tools to detect IR and predict ASCVD and mortality risks in FH population. As demonstrated in several large clinical studies, TyG index is associated with the development and prognosis of cardiovascular diseases [26, 27]. In a prospective study including a total of 1655 nondiabetic patients with acute coronary syndrome with LDL-C below 1.8 mmol/L, a high TyG index level (≥ 8.33) was associated with a higher incidence of acute myocardial infarction (21.2% vs. 15.2%, p = 0.014), larger infarct size (described by cardiac injury biomarkers), and higher incidence of revascularization (8.9% vs. 5.0%, p = 0.035) [38]. In another study focused on elderly acute coronary syndrome patients, TyG index increased by 28% (95%CI 1.06–1.56, *p* for trend = 0.02) for each SD increase in the TyG index. Herein, we found that high TyG index acted as an independent risk factor of ASCVD and mortality in FH adults. To be specific, the risk of ASCVD, all-cause death and cardiovascular death increased by 74% after adjustment (95%CI 1.15–2.63, *p* = 0.01), 55% (95%CI 1.10–2.18, *p* = 0.01) and 79% (95%CI 1.04–3.09, *p* = 0.04) for every 1 unit increase of TyG index after multivariable adjustment. These results indicated that IR plays an essential, detrimental role in FH patients, which could be another explanation for the residual risks of FH. Evaluation and treatment of IR should also be emphasized since most of current therapies focus on the management of LDL-C [35]. Besides, we also noticed that the combination of TyG index and the traditional model led to significant improvements in Cox prediction models of both survival from all-cause mortality (0.483 *vs*. 0.489, *p* = 0.042) and cardiovascular mortality (0.397 *vs*. 0.408, *p* = 0.042).

Surprisingly, a strong U/J-shaped relation was observed according to the RCS results (*p* = 0.0083 for all-cause death and 0.0046 for cardiovascular death, respectively) and the moderate TyG index group had the lowest risk of mortality. When compared with the moderate TyG index group, the low TyG index group had a trend toward an increased risk of all-cause and cardiovascular mortality (HR, 1.19; 95%CI 0.71–2.00 and HR, 2.19; 95%CI 0.96–4.97, respectively), despite no significant differences. A potential cause of the phenomenon is the effect of certain parameters which could not be adjusted, such as hypoglycemia. TyG index was significantly correlated with blood glucose (r, 0.462; *p* < 0.001). The low TyG index group had a trend of worse prognosis, which may be caused by lower blood glucose. Nevertheless, we failed to provide robust statistical evidence on the elevated risk of mortality in the low TyG index group compared with the moderate TyG index group mainly due to the limited sample size.

The current study has several limitations to be noted. Firstly, the small sample size may have limited the statistical power to detect some associations as significant when comparing different groups, as mentioned above. However, up to 114 months of median follow-up duration helps to improve statistical efficiency. Secondly, the data of TyG index was obtained only at baseline and it’s hard to control for possible changes in blood glucose and TG during the follow-up in theory. However, it’s still considered a valid method to evaluate the long-term effects of TyG index according to a large number of reports [32, 39, 40]. Thirdly, the cut-off of the TyG index in this report was based on the RCS results. Therefore, more investigations based on other populations are required to explore whether the 8.5/9.0 cut-off is universal. Lastly, although the adjustment model incorporated the most available demographic and clinical parameters, some residual or unmeasured confounding variables such as laboratory results related with thrombogenesis and coagulation could have affected the results.

Conclusively, results in this pilot study suggested that TyG index was applicable to reflect glucose metabolism status in FH adults, and a high TyG index was an independent risk factor of ASCVD and all-cause mortality in the same population.

## Data Availability

All data generated or analyzed during this study are included in this published article.
